# Early life vitamin D depletion alters the postnatal response to skeletal loading in growing and mature bone

**DOI:** 10.1371/journal.pone.0190675

**Published:** 2018-01-25

**Authors:** Stephanie A. Borg, Harriet Buckley, Robert Owen, Ana Campos Marin, Yongtau Lu, Darryl Eyles, Damien Lacroix, Gwendolen C. Reilly, Tim M. Skerry, Nick J. Bishop

**Affiliations:** 1 Academic Unit of Child Health Department of Oncology & Metabolism, University of Sheffield, Sheffield, United Kingdom; 2 INSIGNEO Institute of in silico medicine, Department of Materials Science and Engineering, University of Sheffield, Sheffield, United Kingdom; 3 Department of Engineering Mechanics, Dalian University of Technology, Dalian, China; 4 Queensland Brain Institute, University of Queensland, Brisbane, QLD; Queensland Centre for Mental Health Research The Park Centre for Mental Health, Wacol QLD, Australia; 5 Academic Unit of Bone Biology, Department of Oncology & Metabolism, University of Sheffield, Sheffield, United Kingdom; 6 Academic Unit of Child Health, Department of Oncology & Metabolism, University of Sheffield; Sheffield Children's NHS Foundation Trust, Sheffield, United Kingdom; Indiana University Purdue University at Indianapolis, UNITED STATES

## Abstract

There is increasing evidence of persistent effects of early life vitamin D exposure on later skeletal health; linking low levels in early life to smaller bone size in childhood as well as increased fracture risk later in adulthood, independently of later vitamin D status. A major determinant of bone mass acquisition across all ages is mechanical loading. We tested the hypothesis in an animal model system that early life vitamin D depletion results in abrogation of the response to mechanical loading, with consequent reduction in bone size, mass and strength during both childhood and adulthood. A murine model was created in which pregnant dams were either vitamin D deficient or replete, and their offspring moved to a vitamin D replete diet at weaning. Tibias of the offspring were mechanically loaded and bone structure, extrinsic strength and growth measured both during growth and after skeletal maturity. Offspring of vitamin D deplete mice demonstrated lower bone mass in the non loaded limb and reduced bone mass accrual in response to loading in both the growing skeleton and after skeletal maturity. Early life vitamin D depletion led to reduced bone strength and altered bone biomechanical properties. These findings suggest early life vitamin D status may, in part, determine the propensity to osteoporosis and fracture that blights later life in many individuals.

## Introduction

Lifelong bone health is determined by exposure to factors from preconception to older age. Bone mass is accrued through childhood and peaks in early adulthood; determinants of this peak include genetic factors and environmental exposures such as diet and physical activity[1]. Osteoporosis is a skeletal disorder characterised by increased bone fragility and fracture. Currently one in three women and one in five men aged 50 years and over experience an osteoporotic fracture; hip fractures are associated with a 36% increased mortality in the first year after fracture[2].

Vitamin D, synthesised in the skin and ingested in the diet, plays a significant role in bone growth and development. During pregnancy 25(OH)D is transferred from the mother to the foetus via the placenta and at birth umbilical cord levels are approximately 80% of maternal levels [3]. Cohort studies have demonstrated that lower maternal Vitamin D status is associated with decreased femur volume[4] and femoral metaphyseal splaying[5] at 34 weeks and decreased bone mineral content[6] and lower bone mass relative to body weight at birth[7]. In addition, lower maternal vitamin D status is associated with reduced bone mineral content and area persisting into childhood[8] and young adulthood[9]. In contrast, infants with greater birth weight have higher bone mass and reduced risk of hip fracture in adult life [10, 11]. In a recent prospective randomised controlled study, maternal vitamin D supplementation increased bone mass at birth in winter-born infants. [12] Previous epidemiological work suggests a relationship between season of birth, and hence maternal sunlight exposure and cutaneous vitamin D synthesis, and hip fracture risk in older people[13].

Although bone remains responsive to mechanical stimulation as an anabolic agent throughout life, bone loss exceeds bone gain with increasing age, suggesting that either the degree of mechanical stimulation, or responsiveness to it, is gradually lost. [14]

Considered together, these findings imply that early life vitamin D exposure has effects that persist into childhood, may impact on peak bone mass accrual and influence subsequent bone loss, the development of osteoporosis and the risk of life-limiting fracture.

**Fig 1:**
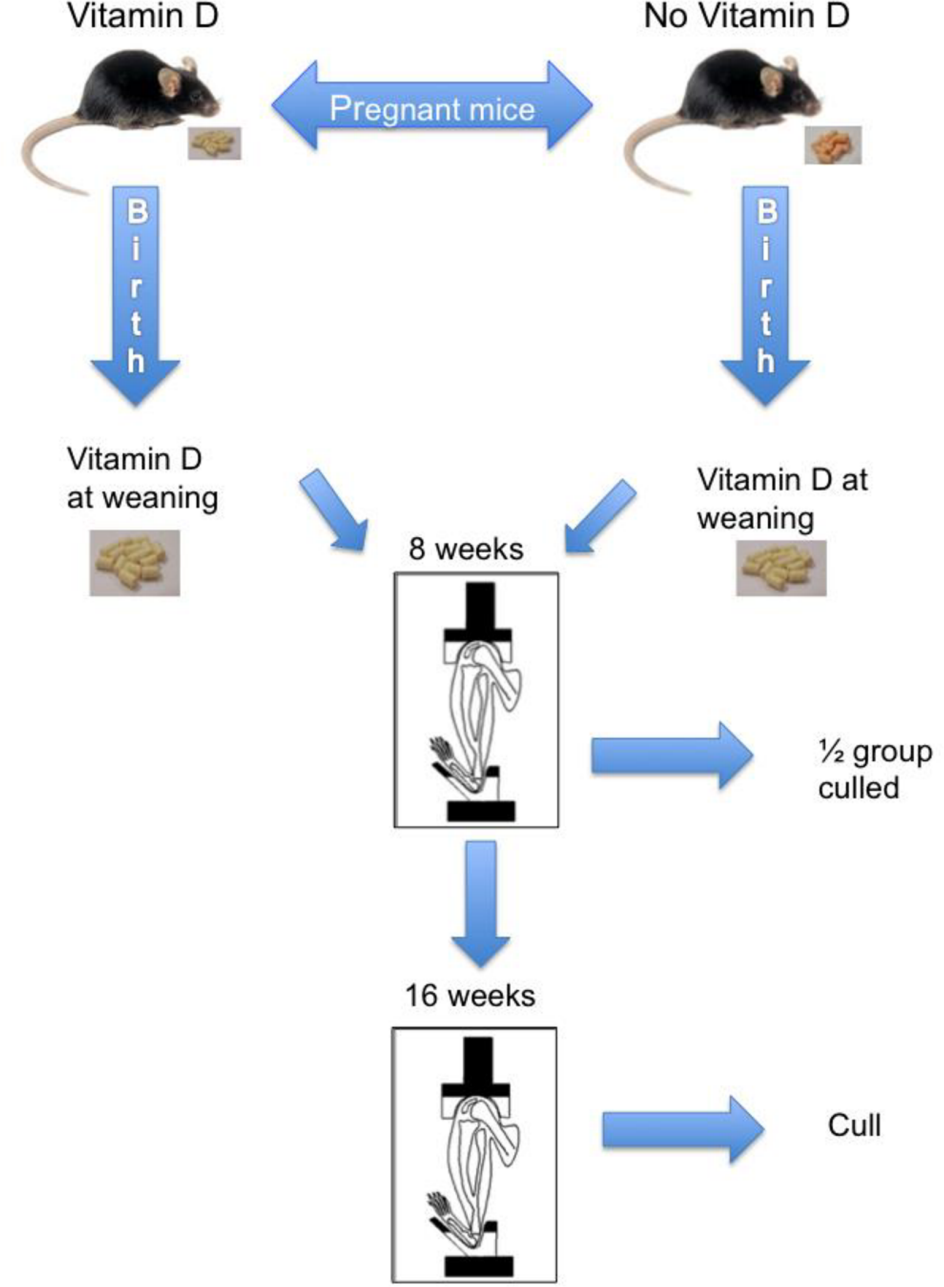
Experimental design showing the creation of murine model of dietary vitamin D deficiency up to culling post mechanical loading.

We hypothesise that antenatal vitamin D status may be responsible for altered bone responsiveness to postnatal mechanical loading, a major anabolic influence on bone size, mass and strength, and thus act as a determinant of later skeletal ill-health. We used C57BL/6 mice to generate vitamin D replete and deplete dams, who went on to deliver litters that remained on the maternal diet until weaning, mimicking the common situation of vitamin D exposure in humans. We applied multiple measurement techniques to assess bone size, mass, strength and activity of lower limbs that had been either subjected, or not, to mechanical loading at 8–10 and 16–18 weeks of age and compared the outcomes both within individual animals, and across groups of animals (Fig 1). The ages were chosen to reflect periods of either continuing or completed skeletal growth and development. In summary we have been able to demonstrate that offspring of vitamin D deplete dams have reduced bone mass and, in response to mechanical loading, accrual of bone mass is further reduced along with a reduction in bone strength.

## Methods

### Animals

A total of 20 breeding females and 96 offspring were used in this study. Four week old C57BL/6 female mice (Charles River, Kent, UK) were purchased and housed at the University of Sheffield Biological Services Unit and had access to food and water ad libitum. All mice were housed in a single room with a 12 hour light/dark cycle at 22 ^0^C under UV filtered (epak Electronics Ltd, UK) fluorescent lighting.

Female mice were fed a Vitamin D supplemented diet (1000 units/kg) or Vitamin D free diet based on AIN93G diet (Research Diets Inc., USA) from four weeks age. Mice were randomly allocated to either Vitamin D replete or deplete diet blindly by technical staff. Investigators were not blinded to study allocation following this. At 10 weeks of age female mice were mated with Vitamin D normal adult males; dams remained on their respective diets throughout gestation until pup weaning. Female offspring remained with their dams until weaning at day 22 whereupon they were weaned onto the Vitamin D replete diet (n = 24). All animals were supplemented with 2 mmol/l Ca^2+^ in their drinking water. After weaning onto the control diet chow all animals were vitamin D replete when mechanically loaded at 10 and 18 weeks (Fig 1).

This study was carried out as per regulations under the Animals (Scientific Procedures) Act 1986. The study was approved by the local committee of University of Sheffield that oversees all animal use within the institution. Loading experiments were performed under isoflurane anaesthesia and blood sampling was performed following cull as per Schedule 1 requirements. All efforts were made to minimize animal suffering.

### 25-OHD3 measurement by LC/MS/MS

25-OHD3 was measured in 3.2mm diameter dried blood spots. Blood was collected by cardiac puncture following cervical dislocation. Spots were rehydrated, extracted and 25-OHD3 derivatised with 4-phenyl-1,2,4-triazioline-3,5-dione (PTAD) according to our published methods[15]. Derivatised samples are reconstituted and analyzed with a API 5000 QTRAP Triple Quadrupole, Linear Ion-Trap LC/MS/MS mass spectrometer (Applied Biosystems, USA) connected to a Dionex Ultimate 3000 LC system according to our published methods[16]. As nearly 90% of 25OHD3 in sera is protein bound, and since this component would be excluded from the red blood cells, it was necessary to obtain the hematocrit fraction and correct 25OHD3 concentrations in sera using the following formula:

Plasma_25-OHD3nM_ = DBS_25-OHD3nM_*1/[1− 0.45 (Adult mouse Hematocrit)] [16].

12 animals per group were studied at each of the two time points following the loading period over two weeks, thus animals were either 10 or 18 weeks old at the point of euthanasia. In each group, bones from the mice either underwent microCT followed by 3 point bending and histomorphometric analysis (n = 6), or underwent microCT (n = 6).

### Mechanical loading in vivo

A non-invasive method of tibial loading was used to examine the responses to mechanical loading at ages 8 weeks or both 8 and 16 weeks (S1 Fig). The peak load of 11N was selected as this is known to induce an osteogenic response in female C57BL/6 mice [17–19]. A 10.5N dynamic load was superimposed onto a 0.5N preload at a rate of 160,000 N/second. Forty trapezoidal-waveform load cycles (0.2 second hold at 11N) with a 10 second interval between each cycle were applied to each mouse’s left tibia for 3 times per week for 2 weeks.

Mice were injected intraperitoneally with calcein (30mg/kg) on the first and last day of loading (D1 and D12). Mice were euthanized on day 15 (where day 1 was the first day of loading). Both left and right tibias were dissected and fixed either in paraformaldehyde (PFA) or frozen wrapped in PBS soaked gauze at -20°C. The contralateral non-loaded limb (right tibia) was treated as internal control for loading (the functional adaptation of the bone is confined to the loaded limb)[20].

### μCT

Fixed tibia were scanned within 48 hours using a SkyScan 1172 desktop μCT machine at a resolution of 4.3μm for morphometric analysis. Frozen tibias were scanned at 10μm for calculation of CSMI with the x-ray source operating at 50kV, 200μA and using a 0.5mm aluminium filter. Two dimensional μCT images were captured and reconstructed by SkyScan NRecon (Bruker, Kontich, Belgium) software at a threshold of 0.0 to 0.16. Trabecular morphometry was characterised by measuring structural parameters in a 1.0mm thick trabecular region, 0.2mm below the growth plate. Cortical morphometry was calculated from a 1.0mm thick region 0.5mm either side of the tibial midshaft. CSMI was determined from a 6mm thick region with the midshaft at the centre. Nomenclature and symbols were used to describe the μCT-derived bone morphometries according to Bouxsein and colleagues [21]. The μCT scans were used for the finite element analysis described below.

### Finite element analysis (FEA)

Whole bone image sets for lower leg bones representative of the loaded and non-loaded groups at each time point were rendered *in silico*, aligned and cropped to remove the growth plates and then the fibula. Voxels were converted to hexahedrons, and FEA was solved using Ansys with the following conditions: the proximal side of the tibia was fully constrained and a unidirectional load of 0.83N in the longitudinal direction of the tibia was applied. The load magnitude of 0.83 N was chosen based on the paper from Prasad et al. (Characterizing gait induced normal strains in a murine tibia cortical bone defect model. Prasad J, Wiater BP, Nork SE, Bain SD, Gross TS. J Biomech. 2010 Oct 19;43(14):2765–70) using only a longitudinal force to avoid excessive bending. However, given that the material properties are linear, the magnitude has little relevance when the objective is to compare the different configurations. A threshold of 25.5% of the maximum grey value was used. The bone was modelled as isotropic linear elastic material with a Young’s modulus of 14.8 GPa and Poisson’s ratio of 0.3.

### Mechanical testing: 3 point bending

After MicroCT measurements, samples were subjected to three point bending analysis using a Bose Electroforce 3200 mechanical testing machine with a 450 N load cell. Bones previously frozen at -20°C in PBS soaked gauze were stored in 0.9% PBS at 4°C overnight prior to testing at 40°C. Rounded supports and press head were used with a constant span support distance of 6 mm for all tibias; bones were positioned with the press head contacting the midshaft. A preload of 0.5 N was applied to the bone before compressing at a constant force of 0.25 N/sec until failure. From the force-displacement curve, stiffness (n/mm) was calculated from the linear elastic region and breaking force from the load at failure.

### Histomorphometry

After μCT analysis, tibias were embedded into LR White Resin. Sections were cut at 10 μm and examined under UV illumination. The bone histomorphometry software Osteomeasure™ was used to measure the double labelled surface, single labelled surface, the separation between the 2 labels and the total bone surface on a 3mm length of endocortical surface and the trabecular surface 0.25mm from the growth plate. Mineralising surface (MS), mineral apposition rate (MAR) and bone formation rate (BFR/BS) were calculated and reported using the nomenclature based on the report of the ASBMR Histomorphometry Nomenclature Committee[22].

### Statistical analysis

All values are expressed as mean ± SD. We determined group sizes by performing a power calculation to lead to an 80% chance of detecting a significant difference (p<0.05). All values use biological replicates and group sizes (n) are 6 throughout where n represents a single mouse. 2 sample Student’s t-test is used throughout for comparison between replete and deplete groups. Nested ANOVA was used to compare loaded vs unloaded within the same animal, accounting for diet during pregnancy; paired Student’s t-test was used to compare the within animal effects of loading for the Figs. P<0.05 was considered to be statistically significant.

Scatterplots were used to describe the entire population without assumptions about the statistical distribution.

## Results

### A lack of dietary vitamin D leads to vitamin D deficiency

Control dams had Vitamin D_3_ levels of 96.1±10.9nM; deficient dams 0.9±0.2nM. Control mice had plasma Vitamin D_3_ levels of 63.0±14.6 nM and 79.2±13.3nM and the early life deplete mice plasma levels 57.6±8.9nM and 58.5±8.9nM at 10 and 18 weeks age respectively following tibial loading. Vitamin D_2_ was detected in 2 samples only with levels below the sensitivity limit (10.7nM)

### Bone structure is negatively impacted by early life Vitamin D deficiency

**Fig 2:**
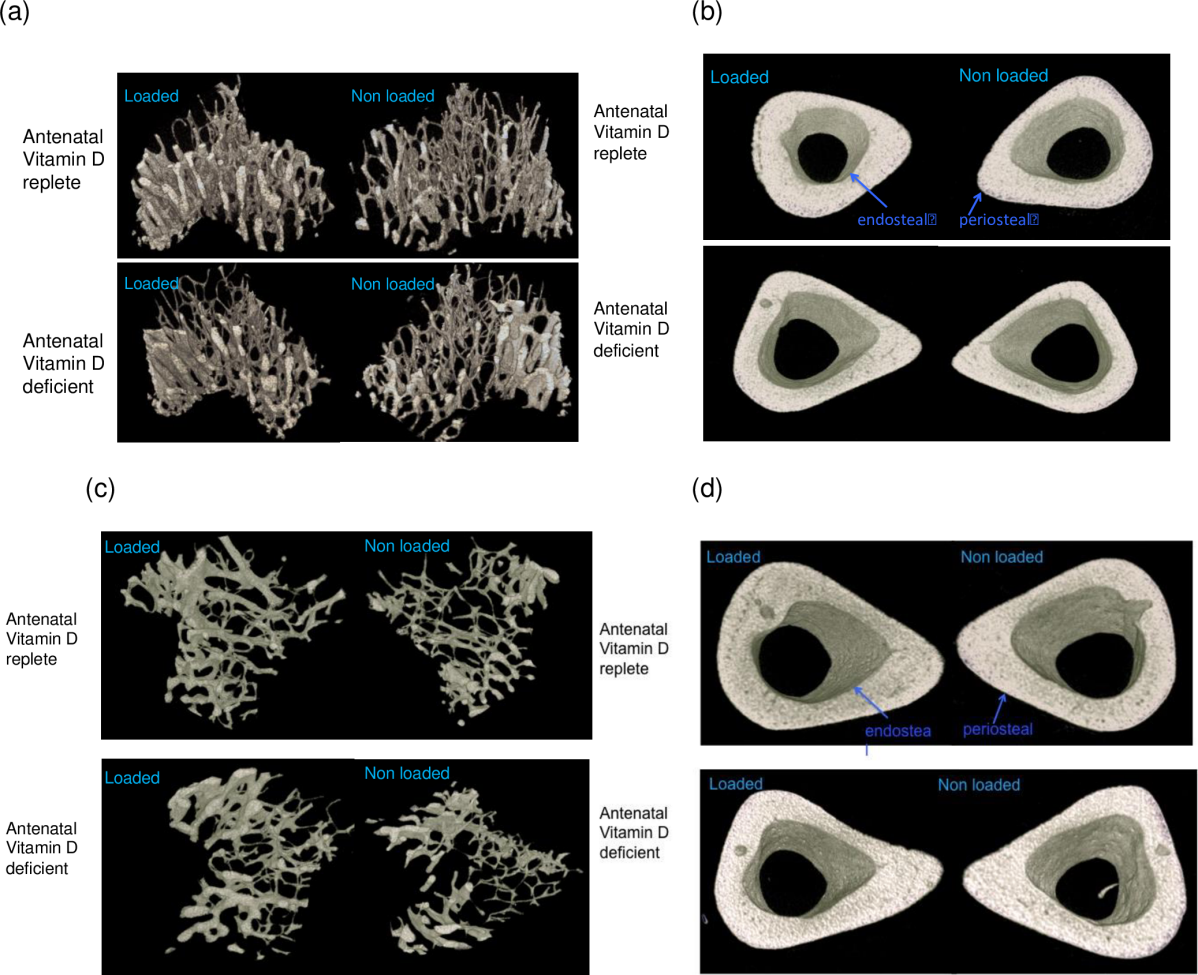
3D reconstructed images of μCT scanned mouse tibia–representative image from each group shown. (a) trabecular region of loaded and non-loaded tibias of early life vitamin D deficient and replete mice aged 10 weeks. (b) mid cortical region of loaded and non-loaded tibias of early life vitamin D deficient and deplete mice aged 10 weeks. (c) trabecular region of loaded and non-loaded tibias of early life vitamin D deficient and replete mice aged 18 weeks. (d) mid cortical region of loaded and non-loaded tibias of early life vitamin D deficient and deplete mice aged 18 weeks.

Fig 2A and 2B shows representative micro-CT images of the non-loaded and loaded cortical and trabecular volumes for mice from the antenatally deplete and replete groups at 10 weeks, and Fig 2C and 2D similar images at 18 weeks.

S1 Table shows the CT-measured parameters for the replete and deplete groups, loaded and non-loaded, at 10 and 18 weeks with percentage differences and estimates of the significance of the difference between the groups, and between the loaded and non-loaded states within individuals.

**Fig 3:**
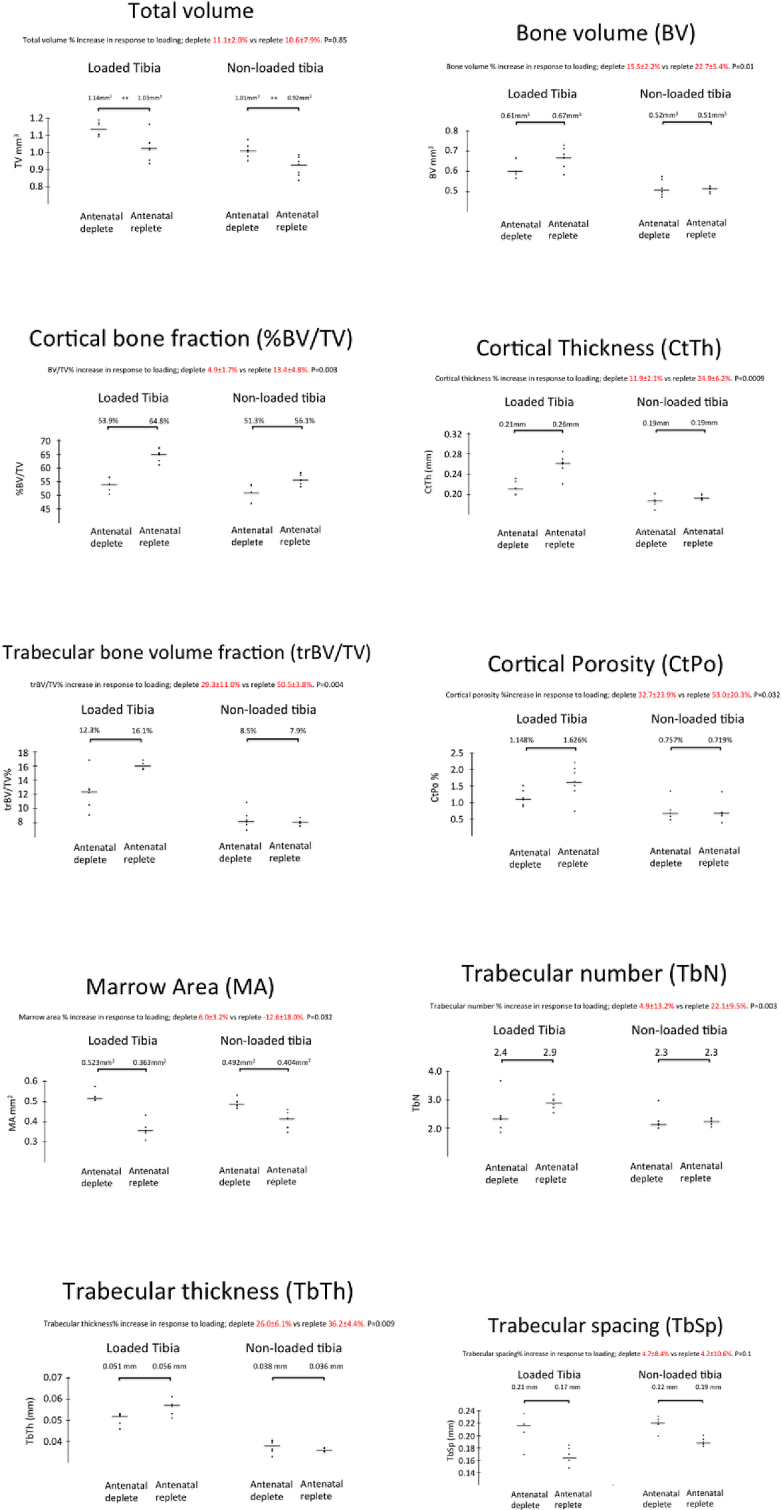
The effect of diet and mechanical loading on the cortical (a) total volume (TV), (b) bone volume (BV), (c) bone fraction (BV/TV), (d) cortical thickness, (e) cortical porosity and (f) marrow area, (g) trabecular bone fraction, (h) trabecular number, (i) trabecular thickness and (j) trabecular spacing at age 10 weeks age. Data are mean ± sd. *P<0.*05*, ** p<0.*01*, *** p<0.*001* by Student *t*-test (n = 6 mice per group for all measured parameters).

At age 10 weeks, cortical tissue volume was greater, bone volume, porosity and thickness similar and bone volume fraction reduced in offspring of Vitamin D-deficient as opposed to control dams without loading. Loading induced increases in tissue volume (Fig 3A), bone volume (Fig 3B), bone volume fraction (Fig 3C), cortical thickness (Fig 3D) and cortical porosity (Fig 2E) in all offspring.

Marrow area (Fig 3F) increased in offspring of deplete dams but was reduced in the offspring of replete dams.

Comparing offspring from replete and deplete dams following mechanical loading, tissue volume was still greater in the offspring of deplete dams (Fig 3A), but bone volume (Fig 3B), bone volume fraction (Fig 3C), cortical thickness (Fig 2D) and cortical porosity (Fig 3E) increased more in offspring of replete dams.

Thus, offspring of deplete dams had initially wider bones and responded to loading by increasing overall tissue volume by 11%, increased cortical thickness by 12% and increased marrow area by 6%, suggesting periosteal apposition (Δ in Fig 2B) and endosteal resorption (↑ in Fig 2B); offspring of replete dams increased tissue volume similarly, but increased cortical thickness by 25% whilst reducing marrow area by 12%, suggesting endosteal as well as periosteal bone apposition.

Trabecular bone volume fraction (Fig 3G), number (Fig 3H), and thickness (Fig 3I) were similar in both groups without loading. Trabecular spacing (Fig 3J) increased in the offspring of vitamin D-deplete as opposed to replete dams without loading. Loading increased bone volume fraction (Fig 3G) and trabecular thickness (Fig 3I) in all offspring, but trabecular number increased (Fig 3H) and spacing decreased (Fig 3J) only in the offspring of replete dams. The increase following loading in bone volume fraction was substantially greater in offspring of replete (50.5%) as opposed deplete (29.6%) dams (Fig 3G). Thus loading induced the formation and retention of more, thicker trabeculae in the offspring of replete as opposed to deplete dams.

**Fig 4:**
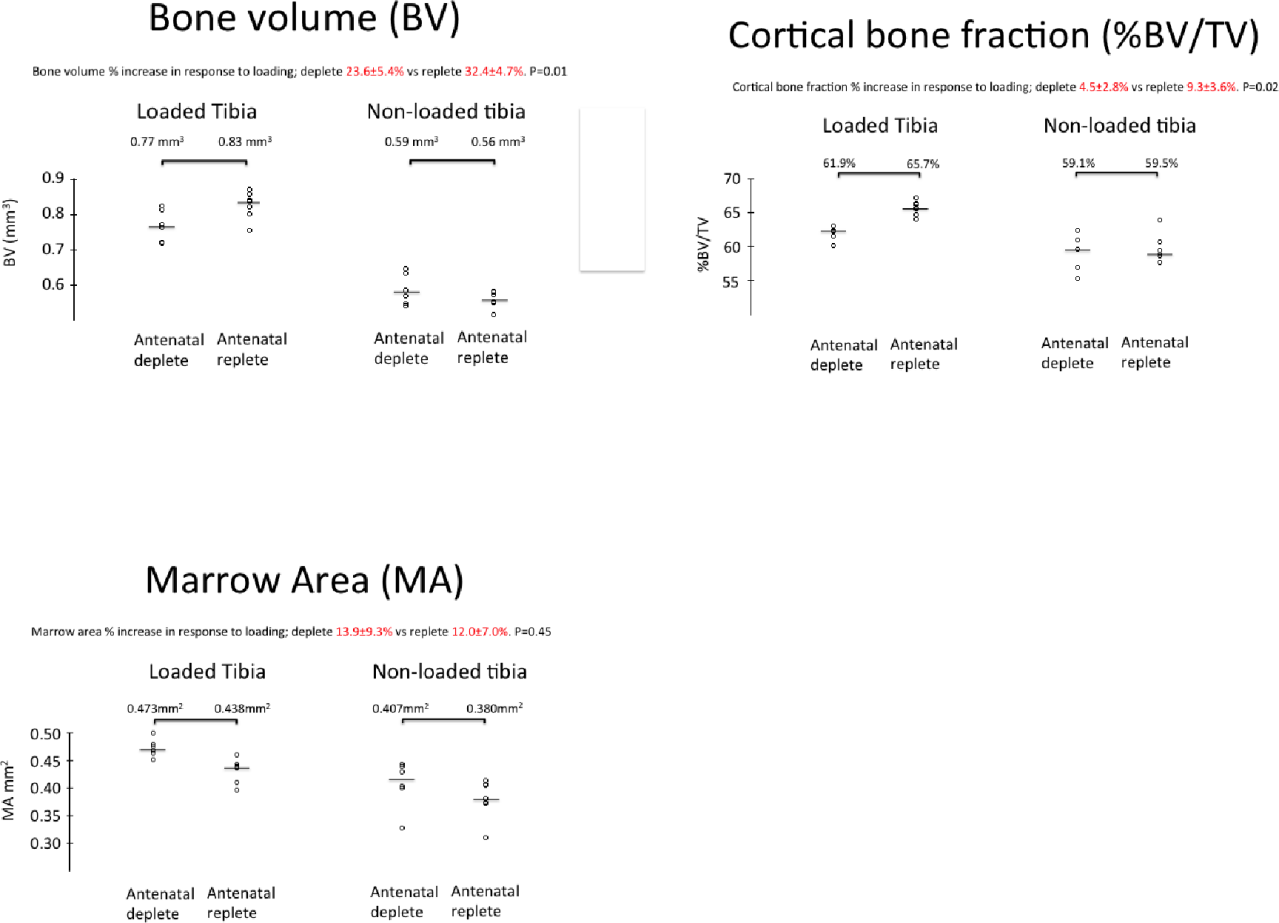
The effect of diet and mechanical loading on cortical (a) bone fraction, (b) bone volume and (c) and marrow area at 18 weeks age. Data are mean ± sd. *P<0.*05*, ** p<0.*01*, *** p<0.*001* by Student *t*-test (n = 6 mice per group for all measured parameters).

At age 18 weeks, parameters for both cortical and trabecular bone were similar between the offspring of vitamin D-deplete and replete dams without loading. In response to loading, in all offspring, cortical tissue volume, bone volume, bone fraction, marrow area, porosity and thickness increased, as did trabecular bone volume fraction and trabecular thickness (Supplementary 3). Cortical bone volume and bone volume fraction increased more and marrow area increased less in the offspring of replete as opposed to deplete dams (Fig 4A–4C). There were no differences between groups for trabecular parameters. Thus at greater age, the effects of antenatal vitamin D depletion on response to loading were evident in cortical bone only, with bone volume fraction increasing approximately twice as much in the replete dams’ offspring.

### Offspring of deplete dams have higher strains across the tibia

**Fig 5:**
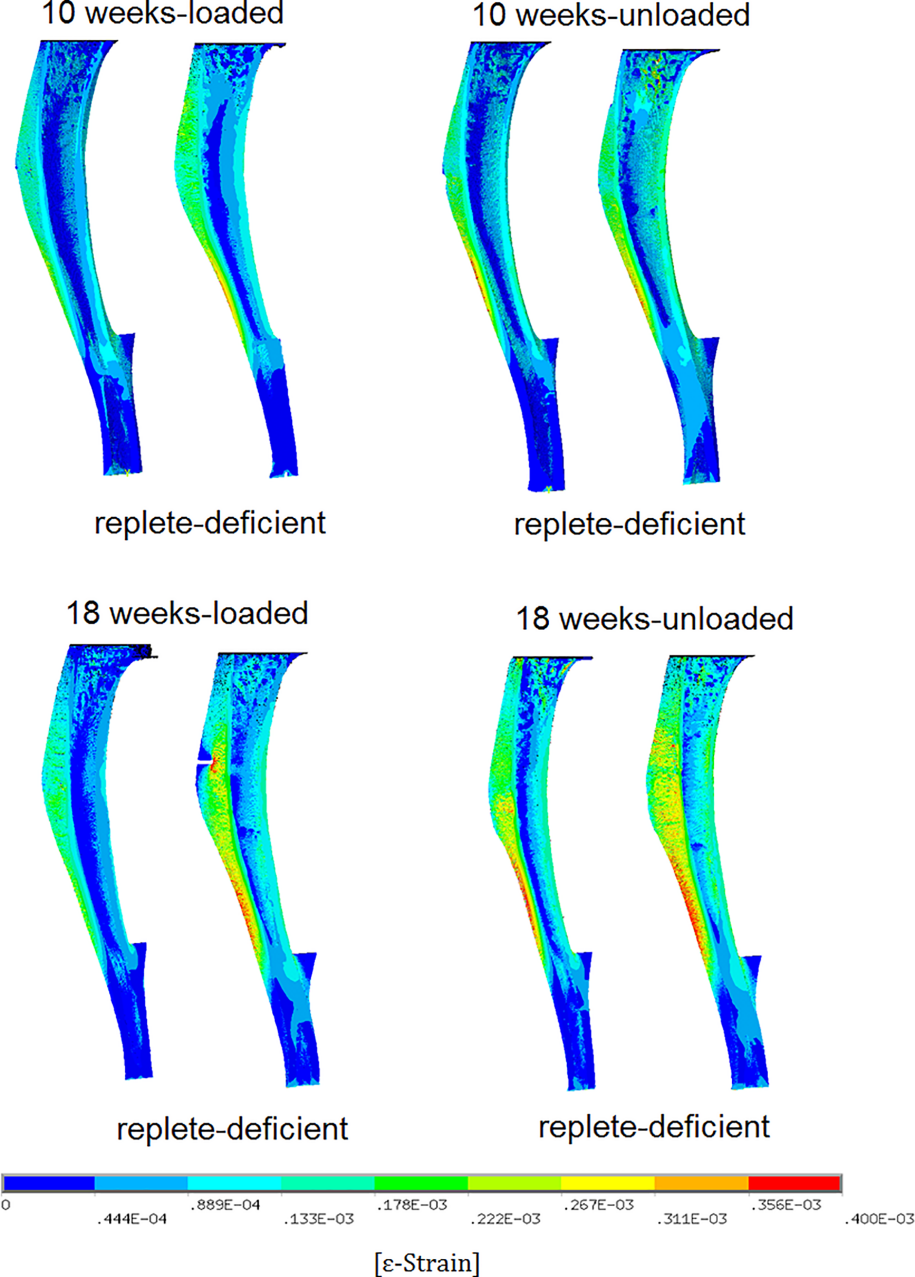
False colour images for whole bone demonstrating the maximum and minimum principal strain at 10 and 18 weeks in previously *in vivo* loaded and non-loaded conditions under an identical load of 0.83N (n = 1 mouse per group).

**Fig 6:**
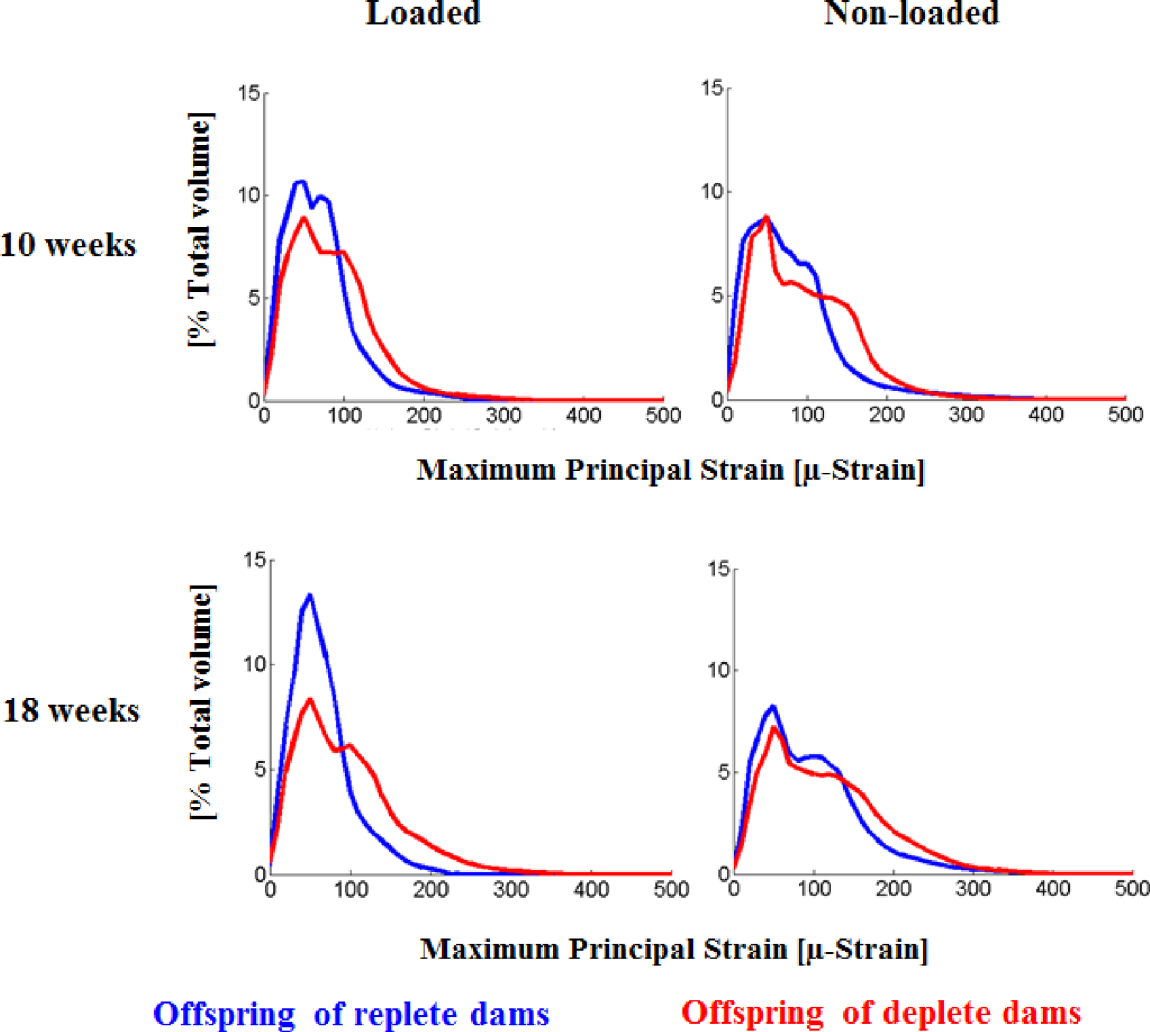
Early life vitamin D replete or deficient comparison of percentage of volume distribution of maximum principal strain distribution at 10 and 18 weeks for loaded and nonloaded bones (n = 1 mouse per group).

Finite element analysis images based on the analysis of representative whole bones are shown in Figs 5 and 6. Fig 5 shows the distribution of maximum principal strain in the cross-section of the whole tibia in all cases. Higher strains were calculated in the offspring of deplete mice but the strain distribution was similar at both ages and loading configuration. Fig 6 shows the volume distribution of maximum principal strain within the whole bone by comparing antenatally vitamin D replete or deplete, and loaded with non-loaded, bones. In the loaded bones in offspring of vitamin D deplete dams, a larger volume of bone had higher strain with a larger difference at 18 weeks than at 10 weeks. Thus bones of offspring of vitamin D deplete dams are less stiff than those of replete dams. This was also observed in non-loaded bones but to a lesser degree. In all groups the non-loaded bone is less stiff, even more so in offspring of the replete dams.

### Tibias in offspring from replete dams are stronger and show improved response to mechanical loading

The results of direct biomechanical testing at 10 and 18 weeks are shown in Table 1. For non-loaded bones at 10 weeks, stiffness and ultimate force (the force needed to break the bone) were higher in the offspring of vitamin D-replete dams; whilst all offspring showed an increase in both parameters in response to loading, that increase was greater in the “replete” group. At 18 weeks, the values for stiffness and ultimate force in non-loaded bones were similar for all offspring; increased stiffness was not shown for the offspring of deplete dams in response to loading, but ultimate force did increase; both stiffness and ultimate force increased for the offspring of replete dams, and the size of increase in both parameters in response to loading was again greater in the offspring of the replete dams.

**Table 1:** Biomechanical data from 3 point bending of mouse tibia for mice at 10 and 18 weeks.

	Stiffness N/mm	Ultimate force N
10 weeks		
**Replete**		
Nonloaded	36.6 (2.5)	8.58 (0.52)
Loaded	43.1 (2.1)	10.95 (0.44)
P for diff	0.0009	<0.0001
**Deplete**		
Nonloaded	33.2 (1.1)	7.74 (0.50)
Loaded	36.1 (2.7)	9.13 (0.75)
P for diff	0.0489	0.0054
P for non-loaded; replete vs deplete	0.0167	0.0169
P for loaded; replete vs deplete	0.0007	0.0008
P for effect of loading by nested ANOVA	<0.0001	<0.0001
18 weeks		
**Replete**		
Nonloaded	38.9 (4.5)	10.60 (1.25)
Loaded	57.4 (8.8)	15.35 (0.95)
P for diff	0.0016	<0.0001
**Deplete**		
Nonloaded	38.6 (6.3)	10.18 (0.46)
Loaded	45.2 (5.6)	11.88 (0.91)
P for diff	0.0807	0.0042
P for non-loaded; replete vs deplete	0.9239	0.4613
P for loaded; replete vs deplete	0.0195	<0.0001
P for effect of loading by nested ANOVA	0.0002	<0.0001

Overall, both the in silico and direct biomechanical testing show that offspring from replete dams had stronger bones that responded more effectively to a mechanical load.

### Mineralising surface is increased in response to loading and decreased in offspring of deplete dams

Histomorphometric analyses representing the extent and rate of new bone formation are shown in S2 Table; because different parts of the bone experience differences in loading, the values for each parameter at the lateral and medial cortices, and the trabecular bone are shown separately. At 10 weeks, there was increased mineralising surface extent at the lateral and trabecular sites for loaded as opposed to non-loaded bones in the offspring of control dams and an increased rate of bone formation in the trabecular compartment. The size of the increase in mineralising surface was significantly greater in response to loading for offspring of replete dams. At 18 weeks, the extent of mineralising surface at the medial and lateral sites was significantly greater in the non-loaded offspring of deplete dams, and fell significantly in the lateral compartment following loading in that group. These results are consistent in respect of the expected increase in trabecular bone formation at 10 weeks.

## Discussion

This study replicates the real life situation of early life vitamin D deficiency in the infant who is born to a Vitamin D deficient mother and then exclusively breast fed until weaning. In this study we have shown that early life vitamin D deficiency leads to decreased bone strength and negatively impacts structure in the bone of growing and mature female mice. Importantly, and for the first time, we have demonstrated that for those with early life vitamin D deficiency, the anabolic response of bone to mechanical loading is reduced in growing bone, affecting both cortical and trabecular bone, with the effect on cortical bone persisting into “adult” life.

In the younger (10 week) mice studied there were baseline differences between the control and early life deficiency group in cortical bone with early life vitamin D deficient mice having wider bones with thinner cortices. This was likely to be an adaptation to reduced bone strength in this group. By 18 weeks when bone growth was complete these differences had disappeared.

We have shown through multiple means that the bones of the offspring of vitamin D replete as opposed to deplete mice are intrinsically stronger and better able to respond effectively to mechanical stimulation. The apparent “uncoupling” of endosteal and periosteal bone formation in the offspring of deplete as opposed to replete dams may give indications as to which specific pathways are being activated to create these important changes; this will be the subject of future studies.

That these changes are present in mice that, following weaning, are vitamin D replete throughout life indicates that early life, as opposed to current, vitamin D status is the cause of these differences. The hypothesis that in utero environmental factors experienced by the fetus could lead to long term adult changes in health was proposed by Barker and colleagues 30 years ago[23]. This laid the foundations for the subsequent work of the Developmental Origins of Health and Disease where sub optimal early life exposures, including maternal nutrition, can lead to long term adverse health effects[24].

Our findings suggest a nutritional programming effect of vitamin D on growing and adult bone. These findings are consistent with multiple data sources that link early life vitamin D status to later bone size[4, 8, 9, 25], structure[6, 26], mass[8, 9, 26, 27] and risk of fracture[9]. They also imply that later correction of vitamin D deficiency does not restore bone’s responsiveness to mechanical stimulation.

There are no similar previous experiments of this nature (i.e combining vitamin D depletion with mechanical loading) with which to compare our data.

The effect of maternal vitamin D deficiency on bone (without loading) has been studied in a range of animal models. Pigs exposed to maternal deficiency showed lower BMD and BMC and decreased biomechanical properties at 8 weeks old[28]. Male and female rats exposed to maternal deficiency but replete from birth showed changes in trabecular thickness at 20 weeks age[29]. Guinea pig pups at birth show reduced long bone BMD and BMC in deplete compared to replete mothers with no change in bone strength[30]. At 28 days when supplemented with vitamin D they maintained lower BMC and strength[31].

Male mice have been previously shown to have no difference in bone strength when on a low Vitamin D diet until weaning[32]. This is in contrast to our study (using only female offspring) where the early life deficiency led to lower stiffness and force required to fracture. In a separate study, maternal deficiency until mid lactation with male pups placed on an obesogenic diet at weaning showed improved trabecular architecture for those on high compared to low vitamin D diet but no differences in strength[33].

The MAVIDOS study, a randomised controlled trial of vitamin D supplementation to pregnant women recently reported changes in BMC in babies born during the winter months.[12] Further follow-up of that cohort may provide the opportunity to test the response of bone to mechanical loading in growing and eventually adult humans.

We used female offspring only in this study. The study with Lanham et al showed greater differences between groups in female compared to male offspring[29]. A maternal low protein diet leads to long changes to bone architecture in female offspring but not male[34]. Mechanical loading has also been shown to demonstrate a greater response to in female compared to male mice[35].

The mice in this study underwent a 2 week regime of mechanical loading of the left tibia at either 8 weeks only or at both 8 and 16 weeks of age. In vivo axial mechanical loading is an effective method to produce a sustained anabolic response[18, 36, 37].

There are no reported studies of mice receiving 2 rounds mechanical loading at separate time points. The loading system used here provides supraphysiological loads over and above loads achieved through regular cage activity. It may be that the trabecular area is maximally stimulated from the first loading. A limitation of this study is that there is not an 18 week age group of mice loaded only at that time point. A further limitation to this study is that the diet used creates a model of complete vitamin D deficiency that is not typical of the human population.

A further limitation of this study is that the loads applied to the bones were the same despite the differences between the groups in predicted stiffness (from FEM) and measured stiffness, both of which showed that the bones from the deplete group were more compliant than the replete group. We believe that our data are robust and that our inferences of a reduced effect of loading in offspring of vitamin D deplete animals stand because the increased compliance of the bones in the deplete group means that the strain stimulus induced by the load of 10.5N was greater than in the replete group. As we show a reduced response to loading of the deplete animals to this increased strain stimulus, we believe that our data represent an under-estimate of the magnitude of the effect we have detected. It could be suggested that strain gauge data would provide useful input to load/strain calibrations for such studies. In other research we and others have used data from strain gauge studies to perform load/strain calibrations ex-vivo, but in mice, we believe that the technique is subject to significant technical limitations. The application of a strain gauge on a foil substrate has little effect on the stiffness of a large bone, but as the size of the bone decreases, the stiffening effect of the applied gauge rises. In these small bones where the gauge would be applied to the outside of a convex curved part of the structure, they are loaded in tension during axial compression of the tibia, and exert a major stiffening effect.

The objective of the FE model was not to replicate the loading applied to the loaded group of mice or to replicate the exact physiological loading condition. Instead we have applied a longitudinal load of 0.83 N based on Prasad et al. in order to calculate the stiffness of the bone and compare it with the different in vivo groups used in this study. Because the material properties are linear, the actual magnitude is less relevant if the main objective was make a comparison between the groups and determine which group has stronger bone. Therefore, it is not possible to relate the higher induced strain with increased anabolic response in replete mice. Instead it shows the effect of vitamin D response before or after birth on the bone stiffness. If a higher volume of bone is undergoing high strain compared to other groups then this means that the overall tibia is softer than others.

We have not identified the mechanisms as yet to account for the changes seen; a clear line of enquiry is along the lines of epigenetic changes. Xue *et al* identified DNA methylation changes in 2 generations of offspring from dams exposed to dietary vitamin D deficiency throughout gestation and weaning[38]. Whilst this study did not examine bone these changes were seen in both somatic and germ cells. Identification of specific pathways is being pursued with a view to better understanding the contribution of early life vitamin D to bone health in later life and to identify new pathways that could impact on osteoporosis prevention and treatment.

This is the first study to report the results of mechanical loading of bone in mice exposed to vitamin D deficiency. This work thus establishes early life vitamin D deficiency as a factor that can contribute to adult osteoporosis; offers a potential solution–supplementation of vitamin D during pregnancy and infancy; and presents a model system to identify pathways that could be targeted in adult life to prevent or treat osteoporosis, a global health problem.

## Supporting information

S1 Fig Mechanical loading of murine tibia a) actual loading in progress and b) schematic of tibial loading device.
(TIF)

S1 Table Trabecular and cortical outcomes following micro-CT analysis of tibias. Values for loaded and nonloaded tibias from early life replete and deplete offspring aged 10 and 18 weeks; values are mean (SD).
(TIF)

S2 Table Dynamic bone formation parameters for tibias. Showing mineralising surface as a proportion of tibial bone surface MS/BS; mineral apposition rate (MAR); bone formation rate (BFR) for cortical and trabecular bone at aged 10 and 18 weeks.
(TIF)
