# Supplementary figures and images for: Early life vitamin D depletion alters the postnatal response to skeletal loading in growing and mature bone
Source: PLoS One. 2018 Jan 25;13(1):e0190675. doi: 10.1371/journal.pone.0190675 (PMC5784894; doi:10.1371/journal.pone.0190675)

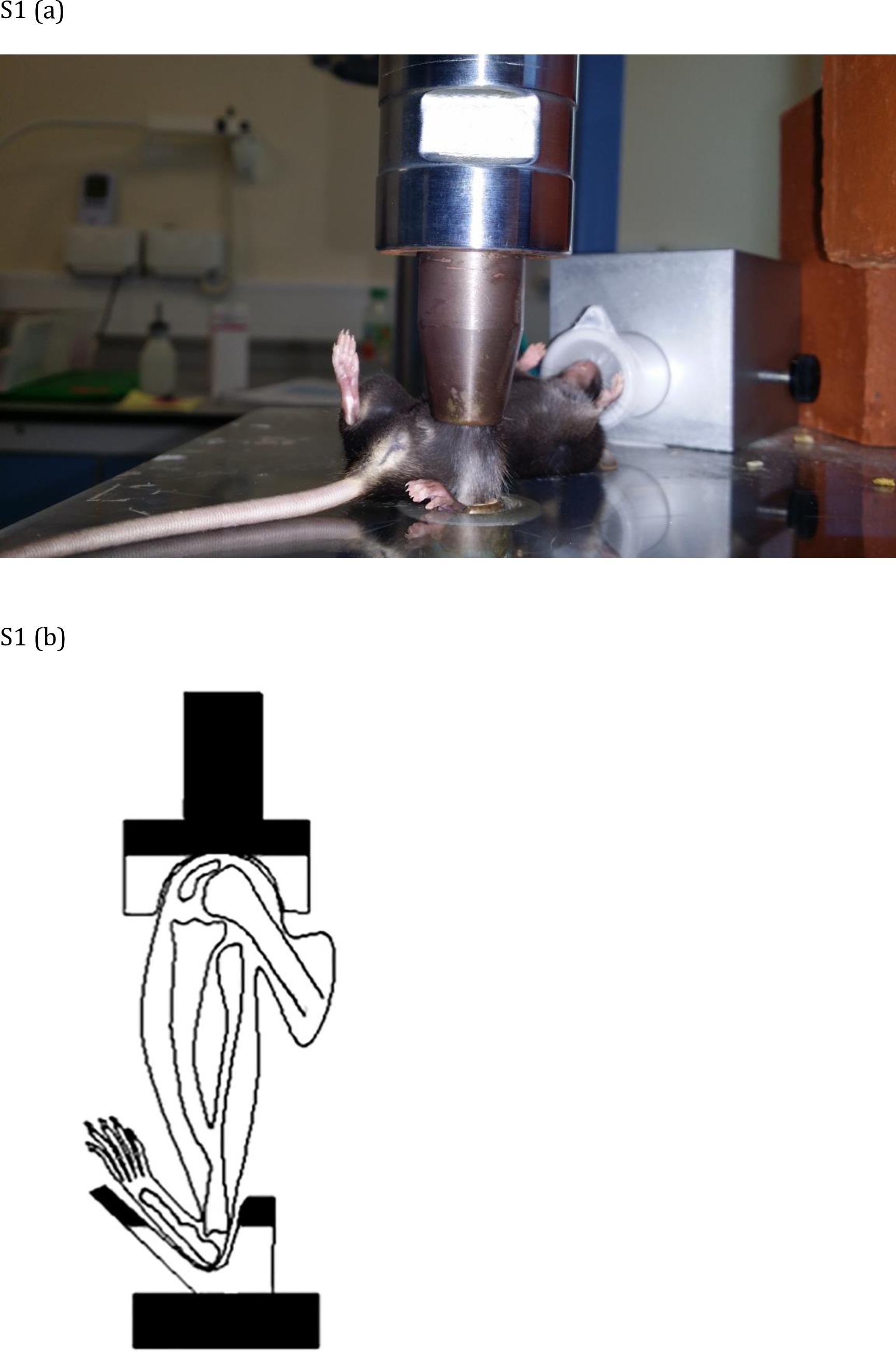

Supplement: S1 Fig — Mechanical loading of murine tibia a) actual loading in progress and b) schematic of tibial loading device. (TIF) [file pone.0190675.s001.tif]

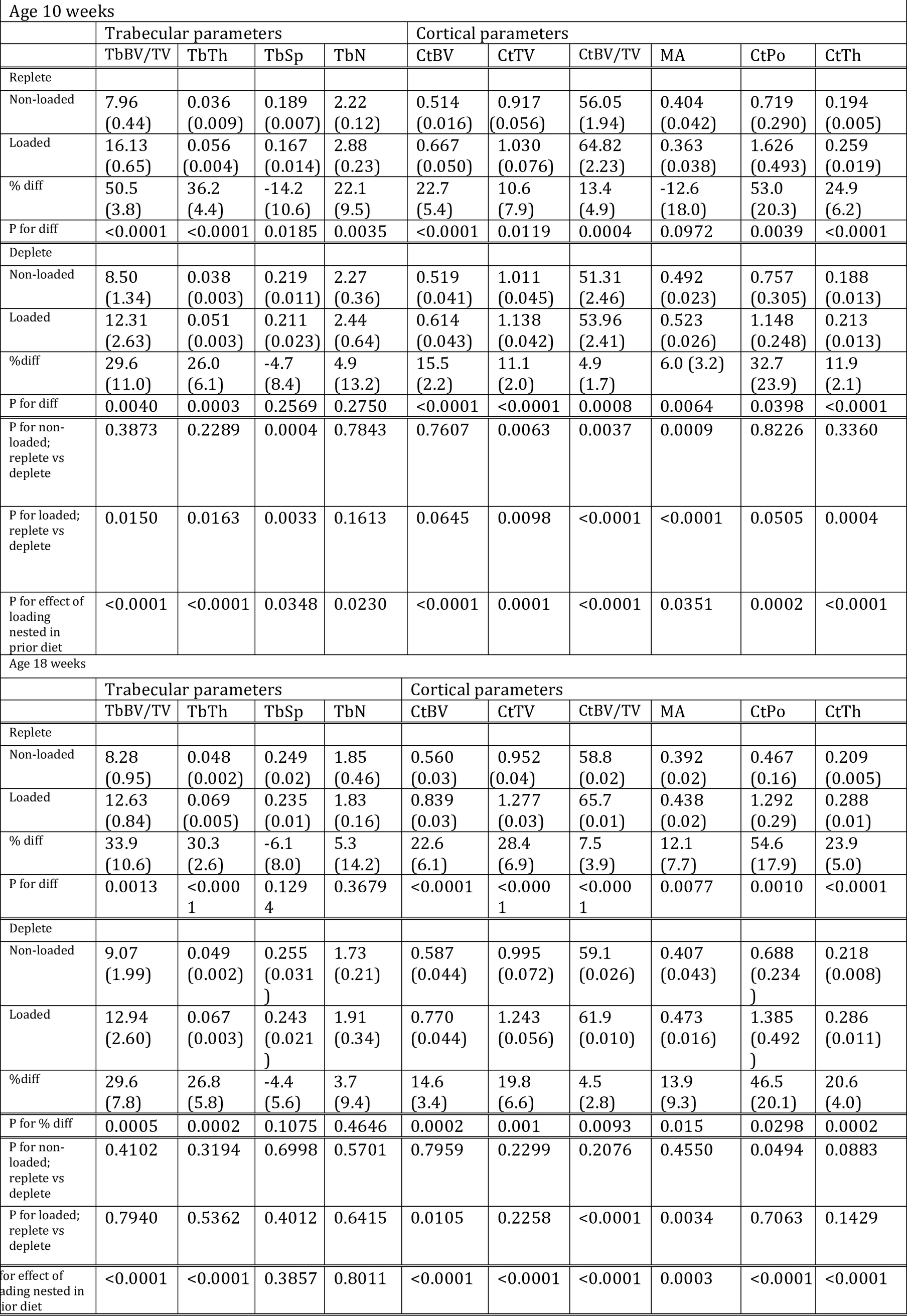

Supplement: S1 Table — Values for loaded and nonloaded tibias from early life replete and deplete offspring aged 10 and 18 weeks; values are mean (SD). (TIF) [file pone.0190675.s002.tif]

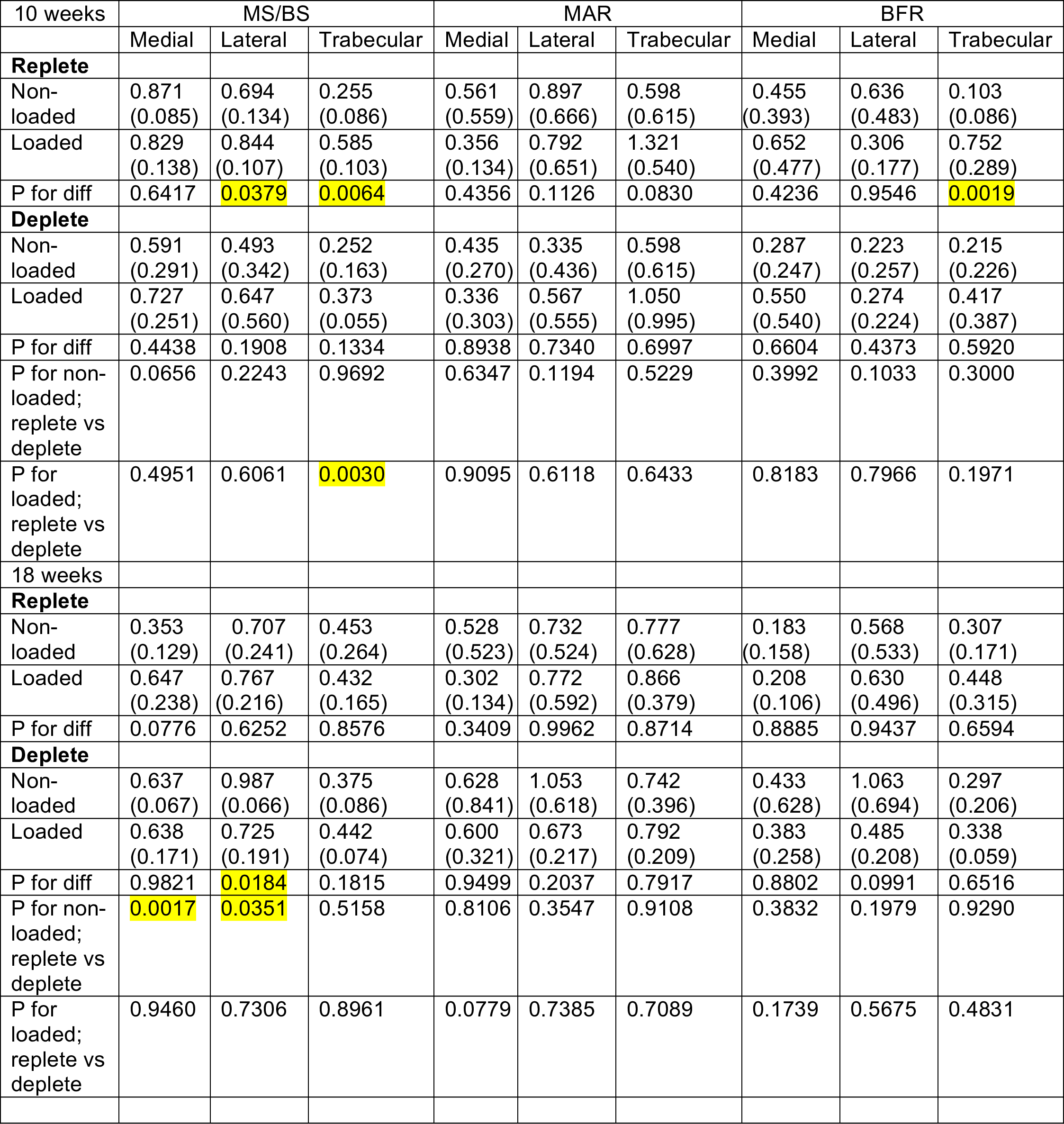

Supplement: S2 Table — Showing mineralising surface as a proportion of tibial bone surface MS/BS; mineral apposition rate (MAR); bone formation rate (BFR) for cortical and trabecular bone at aged 10 and 18 weeks. (TIF) [file pone.0190675.s003.tif]
